# Real-World Use of COMT Inhibitors in the Management of Patients with Parkinson’s Disease in Spain Who Present Early Motor Fluctuations: Interim Results from the REONPARK Study

**DOI:** 10.3390/brainsci15050532

**Published:** 2025-05-21

**Authors:** Lydia López-Manzanares, Juan García Caldentey, Marina Mata Álvarez-Santullano, Dolores Vilas Rolán, Jaime Herreros-Rodríguez, Berta Solano Vila, María Cerdán Sánchez, Tania Delgado Ballestero, Rocío García-Ramos, Ana Rodríguez-Sanz, Jesús Olivares Romero, José Blanco Ameijeiras, Isabel Pijuan Jiménez, Iciar Tegel Ayuela

**Affiliations:** 1 Movement Disorders Unit, Department of Neurology, University Hospital La Princesa, 28006 Madrid, Spain; 2 Neurology Service, Hospital Quirónsalud Palmaplanas and Clínica Rotger, 07010 Palma de Mallorca, Spain; 3 Department of Neurology, University Hospital Infanta Sofia, 28702 Madrid, Spain; 4 Department of Neurology, University Hospital Germans Trias i Pujol, 08916 Barcelona, Spain; 5 Movement Disorders Unit, Department of Neurology, University Hospital Infanta Leonor, 28031 Madrid, Spain; hrinvest@hotmail.com; 6 Department of Neurology, Hospital Josep Trueta i del Santa Caterina de Salt, 17007 Girona, Spain; 7 Department of Neurology, University Hospital Santa Lucia, 30202 Cartagena, Spain; 8 Neurology Service, Hospital Parc Taulí, 08208 Barcelona, Spain; 9 Department of Neurology, Hospital Clínico San Carlos, 28040 Madrid, Spain; 10 Department of Neurology, University Hospital La Paz, 28046 Madrid, Spain; anarods@hotmail.es; 11 Neurology Service, University Hospital Torrecárdenas, 04009 Almería, Spain; 12 Medical Department, Laboratorios Bial, S.A., 28027 Madrid, Spain

**Keywords:** COMT-inhibitors, early fluctuations, OFF time, opicapone, Parkinson’s disease

## Abstract

Objective: We aimed to analyze the real-world use of COMT inhibitors associated with levodopa in patients with Parkinson’s disease (PD) who present early fluctuations and to explore whether early COMT inhibition optimizes treatment outcomes. Methods: REONPARK is an ongoing 2-year prospective observational study. We included patients diagnosed with PD who presented signs of end-of-dose motor fluctuations for <2 years and started COMT inhibitors according to clinical practice. Outcomes included the clinician and patient global impression of change (CGI-C, PGI-C), the Movement Disorder Society-sponsored revision of the Unified Parkinson’s Disease Rating Scale (MDS-UPDRS), the Parkinson’s Disease Questionnaire-8 (PDQ-8), Non-Motor Symptoms Scale (NMSS), 19-Symptom Wearing-off Questionnaire (WOQ-19), and safety. We present a pre-planned interim analysis (cut-off date 3 July 2023) of patients who completed the first 3 months of follow-up. Results: Seventy patients were analyzed (mean levodopa dose at inclusion 484.8 mg; duration of motor fluctuations 0.6 years). In all cases, COMT inhibition was initiated with opicapone, and 81% maintained a stable levodopa dose at 3 months. After 3 months of treatment with opicapone, 73.5% and 62.8% of patients improved on CGI-C and PGI-C, respectively. MDS-UPDRS scores improved significantly with a mean change from baseline of −3.3 ± 7.7 (*p* < 0.001) for Part III and −1.3 ± 1.7 (*p* < 0.001) for Part IV. The mean OFF time decreased from 3.7 ± 2.6 h at baseline to 2.2 ± 2.3 h, and 20.6% of patients no longer experienced OFF periods. Patients experiencing no impact of fluctuations increased from 10% to 45.6%. Conclusions: In PD patients with early fluctuations, three months of opicapone reduced the OFF time and improved functional outcomes, suggesting potential benefits in the early stages.

## 1. Introduction

Parkinson’s disease (PD) is a common neurodegenerative disorder characterized by a progressive loss of dopaminergic neurons in the substantia nigra resulting in striatal dopamine denervation and a hallmark tremor, limb rigidity, and gait and balance problems. However, PD is not just a motor disease but encompasses a variety of psychiatric, autonomic, and sensory symptoms, also affecting psychological and social well-being [1]. Currently lacking a cure, the main goal in treating PD is to improve patients’ functional status and quality of life (QoL).

Levodopa remains the primary and most effective drug for the symptomatic treatment of PD [2]. However, as the disease progresses, the risk of dyskinesias and motor fluctuations increases [3]. Current pharmacological strategies aim to enhance motor function by ensuring a more continuous dopaminergic stimulation, often involving levodopa modifications like dosage increases, more frequent dosing, and sustained release formulations. Although effective in the short term, these approaches do not prevent levodopa trough levels. Importantly, higher levodopa doses have been associated with a high risk of dyskinesia and wearing off [4,5], suggesting pharmacodynamic factors and maladaptive postsynaptic plasticity as underlying mechanisms [5].

Furthermore, it is increasingly accepted that motor fluctuations occur even in the early stages of the disease, within the first years of diagnosis, and are underestimated by routine neurological examination [6]. Longitudinal cohort studies have estimated that motor fluctuations will affect over 50% of patients in the first 5 years of diagnosis [7,8] and even 40% in the first 2.5 years [6]. Consequently, there is an unmet need to control Parkinson’s disease symptoms from the early stages of the disease, providing stable dopamine stimulation while maintaining low doses of levodopa.

Catechol O-methyltransferase (COMT) inhibitors are effective adjunct medications for treating motor fluctuations associated with levodopa therapy. By inhibiting the enzyme COMT, involved in the metabolism of levodopa, COMT inhibitors extend the elimination half-life of levodopa and reduce peak-trough variations, contributing to optimizing the dose of levodopa and stabilizing plasma levels [9]. Three COMT inhibitors are currently available for clinical use. Entacapone and tolcapone appeared earlier on the market with ample evidence for the management of end-dose motor fluctuations [10]; however, the moderate reduction in daily OFF time with entacapone and the potentially fatal liver toxicity of tolcapone have conditioned their use. Opicapone, a third-generation COMT inhibitor with a simplified dose regimen, was developed to overcome these limitations and shows long-lasting enzyme inhibition without the toxicity observed with previous agents. The two pivotal BIPARK studies [11,12] demonstrated efficacy in reducing OFF times without increasing troublesome dyskinesias during ON time, which was maintained in the 1-year open-label extensions and translated into benefits in global function in other observational cohorts [13,14,15]. Moreover, it has been demonstrated that opicapone modulates the pharmacodynamics of levodopa, allowing a more stable dopaminergic stimulation and the use of lower doses of levodopa [16].

The late positioning of COMT inhibitors has dominated clinical practice, probably due to their later approval and the constraints of the short half-life or toxicity of earlier/oldest agents, which may have limited early use [9]. Some evidence suggests the benefit of earlier versus delayed initiation of COMT inhibitors in patients with levodopa-related fluctuations [17,18]. Moreover, recent findings have indicated the benefit of adding opicapone at 50 mg to an additional 100 mg of levodopa in reducing OFF times in a cohort with early fluctuations [19].

In this context, REONPARK was designed to evaluate the use and effectiveness of COMT inhibitors when added to levodopa treatment in a large cohort of patients with PD considered to have early fluctuations under real-world clinical practice conditions. Specifically, this study aimed to assess the impact of COMT inhibitor initiation on levodopa dosing requirements, global function status, disease burden, non-motor symptoms, and quality of life. To our knowledge, REONPARK is the first observational study to systematically analyze these outcomes in cohorts with early fluctuations, thereby providing clinicians with clinically relevant data to inform early therapeutic strategies in PD management.

## 2. Materials and Methods

### 2.1. Study Design and Patient Population

REONPARK is an ongoing prospective observational study conducted in Neurology and Movement Disorder Units in Spain. The study started in January 2022, and site selection was expected to remain open for 3 years. Approximately 10 sites per year were expected to participate and provide 10 patients each during the 1-year recruitment period. Recruitment was opened for new sites to join the study until July 2024, with a target of approximately 260 patients in 26 different sites.

The observation period is 2 years, including 5 routine visits at 3 (±1), 6 (±2), 12 (±2), 18 (±2), and 24 (±2) months after initiation with COMT inhibitor therapy (tolcapone, entacapone, or opicapone).

Eligible patients are adults diagnosed with idiopathic PD according to the UK PD Society Brain Bank (2006) or MDS clinical diagnostic criteria (2015), treated with levodopa/dopa decarboxylase inhibitors (DDCIs), with signs of end-of-dose motor fluctuations for <2 years, and scores ≥ 2 for the 19-Symptom Wearing-off Questionnaire (WOQ-19). Patients with any form of Parkinsonism other than idiopathic, who met the criteria for dementia as per the investigator’s judgment, and participants involved in a clinical trial at the time of study enrollment were excluded. The study was designed to reflect clinical practice as closely as possible, so there was no predefined allocation ratio for the three COMT inhibitors. The choice of treatment was at the discretion of the participating neurologist in charge of the patient according to clinical practice and individual patient characteristics.

This pre-planned interim analysis (cut-off date 3 July 2023) included all patients who had completed the first 3 months of follow-up.

The ethics committee of the Hospital Universitario La Princesa (Madrid, Spain) approved the protocol. The study was conducted in accordance with the Declaration of Helsinki and national regulations, and all patients gave written informed consent to participate. The study was registered in the Spanish Registry of Clinical Studies (REec), (https://reec.aemps.es/reec/public/web.html (18 February 2022)) under the registration number 0012-2022-OBS.

### 2.2. Assessments

Demographic, clinical characteristics, and treatment history for PD from the diagnosis, including previous levodopa treatment and adjunct treatment at inclusion, were collected.

The severity of symptoms at baseline, as per the clinician’s judgment, was assessed using the 7-point scale Clinical Global Impression Severity (CGI-S). Clinician and patient global impressions of change in the patient’s overall status were assessed at 3 months by the Clinician Global Impression of Change (CGI-C) and Patient Global Impression of Change (PGI-C) scales, respectively. Disease burden was assessed using the Movement Disorder Society-sponsored revision of the Unified Parkinson’s Disease Rating Scale (MDS-UPDRS) [20]. The full MDS-UPDRS is a 4-part rating scale covering non-motor and motor experiences of daily living (Part I and II, respectively), motor symptoms (Part III), and motor complications (Part IV). Patients’ QoL was determined using the Parkinson’s Disease Questionnaire-8 (PDQ-8) [21], which ranges from 0 to 100 with higher scores indicating worse QoL. The Non-Motor Symptoms Scale (NMSS) [22] was used to determine the severity and frequency of 30 non-motor symptoms across nine dimensions (cardiovascular, sleep/fatigue, mood/cognition, perceptual problems, attention/memory, gastrointestinal, urinary, sexual function, and miscellany). The presence of wearing-off was measured using the WOQ-19 questionnaire [23], which consists of a checklist of 19 symptoms (9 motor and 10 non-motor symptoms) that patients must identify to be present and assess if they improve with the following dose of anti-PD medication.

The safety of COMT inhibitors was assessed by collecting all adverse events (AEs) (serious or non-serious) that occurred or worsened after exposure to the COMT inhibitor agent, including the causality and severity of the AEs.

### 2.3. Statistical Analysis

The usage of COMT inhibitors, when given adjunct to levodopa/DDCI for the treatment of early fluctuations in PD, was assessed by a composite endpoint consisting of the COMT inhibitor initiated; the daily dose and frequency of dosing; daily levodopa dose and changes in levodopa; and other anti-PD therapies prescribed and changes in non-levodopa medications. Descriptive statistics for quantitative data are presented as the mean ± standard deviation (SD) and/or 95% confidence interval (CI), and median with interquartile range (IQR). For qualitative variables, results are expressed as absolute and relative frequencies.

The baseline levodopa total daily dose and levodopa equivalent daily dose (LEDD) were calculated by considering all the anti-PD treatments and daily frequency that patients were receiving at the time of the inclusion. Changes in the total daily dose of levodopa and LEDD after 3 months of COMT inhibition are presented as the mean absolute and relative change. LEDD was calculated using the conversion formula proposed by Jost ST et al. [24].

Scale-based effectiveness secondary endpoints were the absolute change from baseline to 3 months from the MDS-UPDRS, WOQ-19, NMSS, and PDQ-8 overall and domain scores, analyzed using Wilcoxon’s test. The frequency distributions of patients with OFF periods and with an impact on fluctuations classified according to the MDS-UPDRS Part IV, as well as the frequency distributions of patients with symptoms according to the WOQ-19, were also analyzed. A McNemar test was used to compare the impact of fluctuations (MDS-UPDRS Part IV) between baseline and 3 months, while differences in the mean impact of fluctuations were analyzed by Wilcoxon’s test. Safety analyses were descriptive. Missing data were not considered in the analyses, and the level of significance was taken at a *p*-value of 0.05.

Statistical analysis was performed with R v4.2.0 statistical software.

## 3. Results

### 3.1. Patient Characteristics

Seventy patients from 18 Neurology and Movement Disorders Units across Spain were evaluable at data cut-off (July 2023), all of whom had started COMT inhibition with opicapone. The mean age at inclusion was 64.4 ± 10 years (68.6% male). The mean time since PD diagnosis was 4.8 ± 3.1 years, and the time since the onset of motor fluctuations was 0.6 ± 0.6 years. Clinicians rated the disease severity as mild-to-moderate in 50 patients (72%) at inclusion. Patients had been receiving levodopa for a mean of 40 ± 27 months (3.3 years). The mean daily levodopa dose at inclusion was 484.8 ± 212.5 mg, with 63.8% of patients receiving three doses, and the mean LEDD was 665.0 ± 257.4 (Table 1). In total, 55 patients (78.6%) were receiving MAO inhibitors, 41 (58.6%) were receiving dopamine agonists, 6 (8.6%) were receiving anticholinergics and 4 (5.7%) were receiving amantadine.

At baseline, the mean MDS-UPDRS Parts III and IV were 28.6 ± 13.9 and 4.4 ± 1.7, respectively. Based on Part IV, 87.1% of patients reported ON time without any dyskinesia, and the remaining patients (12.9%) characterized their dyskinesias as not being troublesome (i.e., they interfered slightly or minimally with normal functioning). The mean OFF time was 3.7 ± 2.6 h. The mean total WOQ-19 score was 5.1 ± 2.4, and the mean NMSS was 33.3 ± 34.7.

### 3.2. Changes in Levodopa Dosing and Other Anti-Parkinsonian Medications

After 3 months of COMT inhibition, the levodopa dose remained stable in most patients (81.8%) and was adjusted only in 12 patients, of whom 5 had their dose reduced. The mean LEDD was 1017.6 ± 420.5 mg.

During the 3 months of follow-up, an MAO inhibitor (safinamide) and amantadine were initiated in one patient each. At the time of analysis, four patients discontinued opicapone due to AEs (n = 2, one of which switched to entacapone), patient decision (n = 1), or another reason (n = 1) after a mean time of 80 days.

### 3.3. Effectiveness

**Figure 1:**
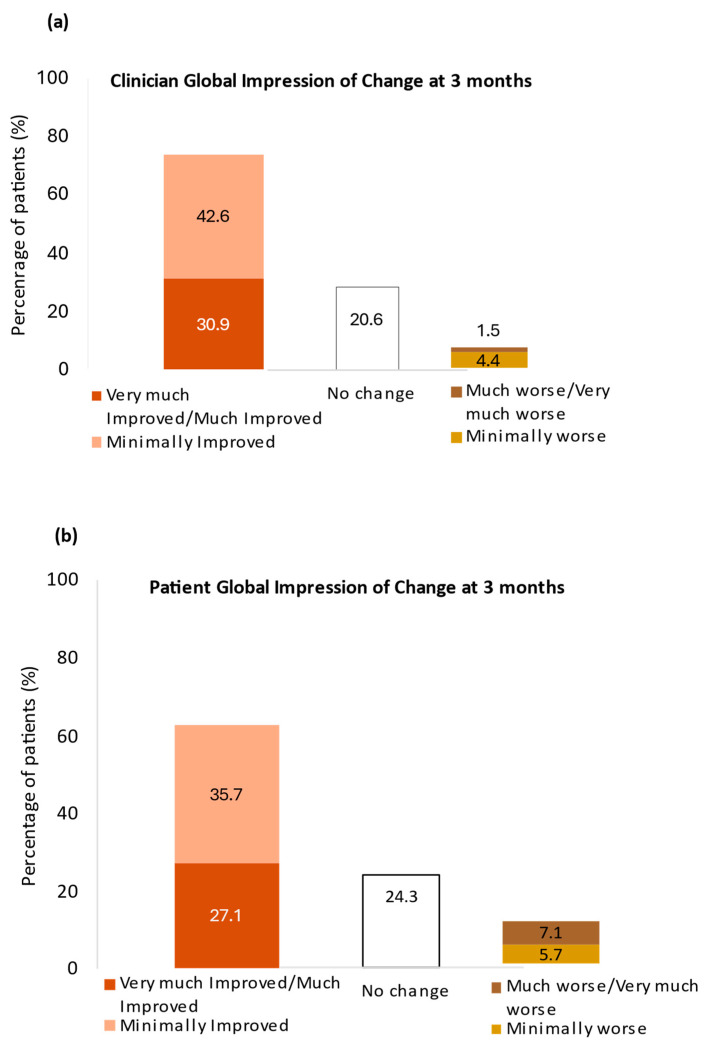
Global impression of change at 3 months of COMT inhibition with opicapone. (**a**) Clinician-rated (CGI-C, n = 68) and (**b**) self-rated by the patient (PGI-C, n = 70). CGI-C and PGI-C are rated on a 7-point Likert scale from 1 = very much improved to 7 = very much worse. CGI-C, clinician global impression of change; PGI-C, patient global impression of change.

After 3 months of COMT inhibition with opicapone, 73.5% of patients had improved, as judged by the clinicians. In total, 62.8% self-reported to have improved after 3 months of treatment with opicapone (Figure 1).

Mean MDS-UPDRS Part III (motor symptoms) scores improved by a mean of −3.3 ± 7.7 (95% CI: −5.2 to −1.5) (*p* < 0.001); Part IV (motor complications) scores improved by a mean of −1.3 ± 1.7 (95% CI: −1.7 to −0.9) (*p* < 0.001); and Part II (motor aspects of ADL) scores improved by a mean of −1.2 ± 4.5 (95% CI: −2.3 to −0.1) (*p* = 0.018) (Table 2). There were no significant changes in the mean MDS-UPDRS for Part I (non-motor aspects of ADL) scores during the first 3 months. Total MDS-UPDRS score improved significantly by a mean of −5.7 ± 11.4 (*p* < 0.001). Based on Part IV, 76.5% of patients reported an ON time without any dyskinesias, and 23.5% characterized their dyskinesias as not being troublesome.

**Figure 2:**
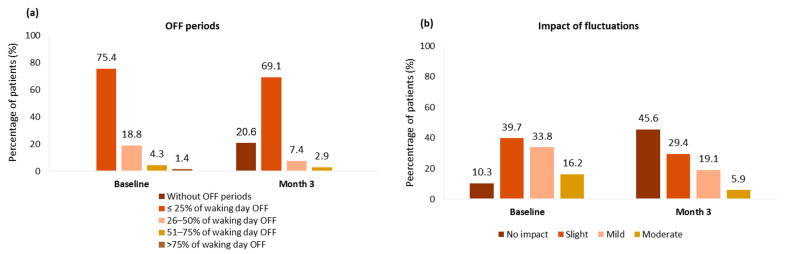
Experience of OFF periods (**a**) and impact of fluctuations (**b**) before and after 3 months of COMT inhibition with opicapone based on Part IV MDS-UPDRS. Missing data: baseline, n = 1; month 3, n = 1. MDS-UPDRS, Movement Disorder Society-Unified Parkinson’s Disease Rating.

The mean OFF time was reduced from baseline to 2.2 ± 2.3 h, and 20.6% of patients no longer experienced OFF periods (Figure 2a). The mean impact of fluctuations decreased significantly from 1.6 ± 0.9 at baseline to 0.9 ± 0.9 at month 3 (*p* < 0.001). The percentage of patients with no impact of fluctuations increased from 10% at baseline to 45.6% after 3 months of opicapone, and the percentage of patients experiencing slight, mild, and moderate impact of fluctuations decreased (Figure 2b). Supplementary Table S1 shows statistical differences in frequency distributions of the impact of fluctuations (*p* < 0.001), with 41 patients (60.8%) improving at month 3.

**Figure 3:**
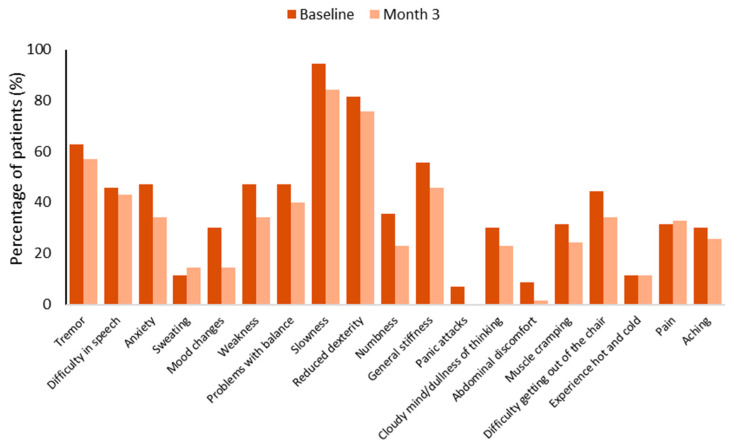
Frequency of symptoms experienced by patients using the 19-items wearing-off questionnaire (WOQ-19).

The mean baseline total WOQ-19 score of 5.1 ± 2.4 was significantly reduced by a mean of 1.3 ± 2.1 (95% CI: −1.8 to −0.8) to 3.8 ± 2.6 after 3 months of COMT inhibition with opicapone (*p* < 0.001). After 3 months, most of the baseline wearing-off symptoms, both motor and non-motor, decreased except for sweating and pain (Figure 3).

**Figure 4:**
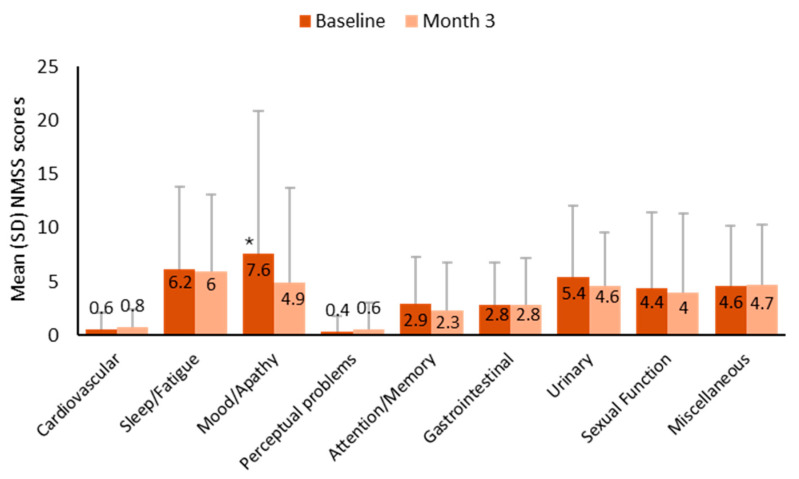
Changes in non-motor symptoms during the first 3 months of COMT inhibition with opicapone. * *p* < 0.05 vs. baseline. NMSS, non-motor symptoms scale; SD, standard deviation. The range of total NMSS scores is as follows: 0–360: 0–20, slight burden; 21–40, moderate burden; 41–70, severe burden; 71–360, very severe burden of symptoms.

The NMSS total score was reduced (i.e., improved) by a mean of 4.6 ± 19.9 (95% CI: −10.9 to 1.8), from 33.3 ± 34.7 at baseline to 28.7 ± 31.0 at 3 months (*p* = 0.207). By domains, only mood/apathy showed a significant change from baseline, with a mean reduction of 2.7 ± 9.3 points (95% CI: −5.2 to −0.2) (*p* = 0.006) (Figure 4). The PDQ-8 total score decreased from 6.2 ± 5.2 at baseline to 5.7 ± 4.9 at 3 months, although the change was not significant (*p* = 0.422).

### 3.4. Safety

In total, 27 of the 70 patients (38.6%) experienced 69 AEs, of which 22 were considered related to opicapone and graded as mild-to-moderate. The incidence of dyskinesia, nervousness, restlessness, and somnolence was >2% (Supplementary Table S2). Two patients experienced a serious AE (SAE), neither of which was related to opicapone treatment.

## 4. Discussion

The findings of this pre-planned 3-month interim analysis provide an early assessment of the study outcomes and should be interpreted in the context of the interim nature of the data. In addition, by the time the database was locked, all the evaluable patients had started treatment with opicapone. Therefore, until the final results are available, we will not have a comprehensive analysis of the real-world, long-term use of COMT inhibitors for the treatment of early motor fluctuations in PD patients.

This analysis showed that in patients diagnosed with PD and with early motor fluctuations (0.6 years), treatment with opicapone for 3 months had beneficial effects with a manageable safety profile. In this cohort of patients with PD showing early fluctuations, opicapone effectively reduced the OFF time and the impact of fluctuations and increased the ON time without troublesome dyskinesias. Moreover, opicapone was able to completely avoid OFF periods in 20.6% of patients who reported no OFF time at all after 3 months of treatment. These observations are in line with the results of BIPARK phase 3 trials in more advanced patients [11,12]. Patients from BIPARK studies had a higher mean baseline OFF time (~6 h) and daily levodopa dose (~700 mg) compared to 3.7 hs and 484 mg, respectively, in REONPARK. This is noteworthy because it suggests that PD patients may benefit from COMT inhibition with opicapone from showing very early fluctuations. In support of this, there are the post hoc analyses of BIPARK I and II in patients at earlier and later stages of the development of motor fluctuations and levodopa treatment. Their results showed the efficacy of opicapone in reducing the OFF time and ON time without troublesome dyskinesias over the whole trajectory of motor fluctuation evolution and suggested, in particular, an enhanced efficacy when used in earlier stages [18]. The severity of OFF time, together with the duration, has an impact on daily functioning and the QoL of PD patients [25]. The results of reducing the impact of fluctuations with opicapone, although preliminary and conservative, might suggest potential contributions to QoL from the early introduction in the course of the disease.

Most patients remained on a stable dose of levodopa, and five patients had their dose reduced, supporting the contribution of opicapone in sustaining overall lower doses of levodopa. The benefits of adding opicapone to levodopa therapy were well-perceived by physicians and patients themselves, with 73.5% and 62.9%, respectively, reporting clinical improvement in the global PD condition. These percentages are similar to those reported in pivotal studies (73% and 72.1%, respectively) [11] and in more advanced real-life cohorts where the majority of clinicians (≈72%) and patients (≈78%) noticed improvement after 3 months of treatment [13,15]. The final results will show whether these rates of global change continue in the long term.

This interim analysis also showed an improvement in motor control, as evidenced by the significant change in MDS-UPDRS Parts II, III, and IV. Importantly, the observed change of −3.3 for Part III and −1.3 for Part IV reached the threshold of 3.25 points and 0.9 points, respectively, which is accepted as a clinically meaningful improvement [26,27]. Thus, opicapone may be effective in improving the quality of ON times in PD patients with motor fluctuations. It is also noteworthy that the absolute change seen in Part III after 3 months of opicapone use is greater compared to studies of advanced-stage disease with similar follow-up [12,15], with the exception of OPTIPARK where the change was −4.6 [13]. The ADOPTION study, conducted in a cohort of patients showing early fluctuations similar to our patients, showed a trend for greater improvements in Part III with opicapone (−3.4) compared with levodopa at 100 mg (−2.5) in the first month and a greater reduction in the percentage of OFF time (*p* = 0.0015) [19].

Most wearing-off symptoms, both motor and non-motor, decreased after 3 months of opicapone use, which is in line with previous observational cohorts with more advanced stages [13,15].

The majority of non-motor symptoms detected in the NMSS remained stable from baseline, except mood/apathy, which improved significantly. When interpreting these results, it should be noted that patients had low NMSS scores at baseline (with a mean of 33.3), indicating a moderate burden of non-motor symptoms (NMSS total score 21–40) [14]). This is important because the probability of improvement in the NMSS appears to be related to the baseline score [28]. Therefore, any significant change or stabilization, even if positive, should be cautiously treated as clinically relevant, especially in the first 3 months of treatment. The fact that most non-motor symptoms were stabilized after 3 months of opicapone adds to the paucity of real-world evidence for the benefit of COMT inhibition with opicapone on non-motor symptoms [13,14,15], which has long been under-recognized. A similar outcome occurred for the patients’ QoL. We observed a nonsignificant improvement in total PDQ-8 scores at 3 months in a population with good health status at baseline, as indicated by the low baseline scores. These results were to be expected, as greater PDQ-8 improvements have been found to be associated with worse baseline PDQ-8 and NMSS scores [29]. However, although the PDQ-8 does not seem to capture specific aspects of QoL in relation to an improvement in symptoms during the first 3 months of treatment, patients did show a favorable perspective on their disease, as indicated by the PGI-C.

Treatment with opicapone was well tolerated, with a rate of drug discontinuation due to AEs of 2.8%, which is significantly lower than that reported in BIPARKs trials (ranging from 4% to 12%) [11,12] and other observational studies (ranging from 6% to 23%) [13,14,15]. This is likely due to the less advanced disease and shorter duration of motor fluctuations in REONPARK compared to previous cohorts of around 8 years of disease course and over 2 years of motor fluctuations [11,12,13,14,15,30]. In fact, the post hoc safety analysis of BIPARK I and II showed a more favorable tolerability profile in patients who were earlier in their disease course and levodopa treatment pathway [30]. All related AEs (27.6%) were mild-to-moderate in severity, and no serious AEs related to the drug were reported. It is also remarkable that no cases of hallucinations were reported in this cohort of patients showing early fluctuations, and no cases of dyskinesia led to discontinuation in contrast to studies in more advanced patients [11,12,13,14,15,19].

One of the main criticisms of this interim analysis is that it only reports data on opicapone, which does not reflect the real-world use of COMT inhibitors for the treatment of early motor fluctuations, which was the main objective of this study. In addition, the results from this pre-planned analysis after 3 months reflect only 12% of the planned 2-year follow-up period, making it difficult to draw definitive conclusions. Also, the effect of COMT inhibition was analyzed in a population of patients with mild or moderate non-motor symptoms and minimally affected QoL; therefore, the results cannot be extrapolated to patients with more severe impairment. The main strength of the study is that it focuses on a PD population showing early fluctuations, which has been the subject of very little research, where opicapone shows similar or even better benefits when used in early compared to more advanced stages.

## 5. Conclusions

This pre-planned interim analysis shows that PD patients with early fluctuations may benefit from opicapone use from a very early stage. Three months of COMT inhibition with opicapone reduced OFF times and increased the percentage of patients without OFF periods and the percentage of patients experiencing no impact from fluctuations. Significant changes in Parts III and IV of MDS-UPDRS, which assess motor symptoms and motor complications, were indicative of clinically meaningful improvement. Treatment with opicapone seems to stabilize mild-to-moderate non-motor symptoms. The safety profile in this cohort of less advanced patients was good and even more favorable compared to those showing late fluctuations.

The final results will shed light on real-world trends in the use of COMTs and their long-term effectiveness and safety in PD patients with early fluctuations, but in the meantime, it is recommended that clinical judgment be reserved.

## Figures and Tables

**Table 1:** Baseline patient characteristics (N = 70).

Characteristic	Value
**Age** (years), mean ± SD	64.4 ± 10
**Sex**, n (%) Male Female	48 (68.6) 22 (31.4)
**Time since PD diagnosis** (years), mean ± SD	4.8 ± 3.1
**Time since onset of motor fluctuations** (years), mean ± SD	0.6 ± 0.6
**Hoehn and Yahr stage**, n (%) I II III	15 (21.4) 49 (70) 6 (8.6)
**PD subtype at diagnosis**, n (%) ^a^ Mild motor-predominant PD Diffuse malignant PD Intermediate	46 (71.9) 5 (7.8) 13 (20.3)
**Clinical Global Impression Severity of illness (CGI-S)**, n (%) ^b^ Borderline Mild Moderate Marked Severe	7 (10.1) 25 (36.2) 25 (36.2) 11 (15.9) 1 (1.4)
History of dyskinesias, n (%)	11 (15.7)
**MDS-UPDRS Part III (motor) score**, mean ± SD	28.6 ± 13.9
**MDS-UPDRS Part IV (dyskinesia) score,** mean ± SD	4.4 ± 1.7
**Off time** Absolute time (hours) Percentage of total awake time (%)	3.7 ± 2.6 22.3 ±15.8
**Wearing-off symptoms (WOQ-19 score)**, mean ± SD	5.1 ± 2.4
**NMSS score**, mean ± SD	33.3 ± 34.7
**Levodopa daily dose at inclusion**, (mg) mean ± SD ^c^ Median (IQR)	484.8 ± 212.5 450 (300–600)
**Number of daily doses of levodopa**, n (%) ^d^ 2 3 4 5 6	3 (4.3) 44 (63.8) 16 (23.2) 3 (4.3) 2 (2.9)
**LEDD**, (mg) mean ± SD ^e^ Median (IQR)	665.0 ± 257.4 605.0 (501.2–775.0)
**Levodopa treatment at inclusion**	
Levodopa and carbidopa (100/25 mg) Levodopa and carbidopa (250/25 mg) Levodopa and benserazide (200/50 mg) Levodopa-controlled release	37 (53.6) 9 (13) 22 (31.9) 5 (7.2)
**Adjunct treatment at inclusion**, n (%)	
MAOI ^¶^ DA ^¶^ Amantadine Anticholinergics (trihexyphenidyl)	55 (78.6) 41 (58.6) 4 (5.7) 6 (8.6)
**Main Comorbidities**, n (%)	
Hypertension Dyslipidemia Depression Anxiety Diabetes	16 (22.9) 9 (12.9) 6 (8.6) 3 (4.3) 3 (4.3)

Missing data (^a^ n = 6; ^b^ n = 1, ^c^ n = 1, ^d^ n = 1, ^e^ n = 2). ^¶^ MAOI: rasagiline, n = 31; safinamide, n = 24. DA: pramipexole, n = 28; ropirinole, n = 8; rotigotine, n = 5. DA, dopamine agonists; IQR, interquartile range; LEDD, levodopa equivalent daily dose; MAOI, monoamine oxidase inhibitors; MDS-UPDRS, Movement Disorder Society-sponsored revision of the Unified Parkinson’s Disease Rating Scale; NMSS, Non-Motor Symptoms Scale; PD, Parkinson’s disease; SD, standard deviation; WOQ-19, 19-Symptom Wearing-off Questionnaire.

**Table 2:** Changes in MDS-UPDRS scores from baseline to 3 months using COMT inhibition with opicapone.

	Baseline	3 Months	Absolute Change	Percentage of Change	*p*-Value
**Part I** (non-motor experiences of daily living)	7.5 ± 5.3 (95% CI: 6.2 to 8.7)	7.6 ± 6.0 (95% CI: 6.2 to 9.1)	0.2 ± 4.2 (95% CI: −0.8 to 1.2)	6.9 ± 66.7 (95% CI: −9.3 to 23)	0.956
**Part II** (motor experiences of daily living)	8.8 ± 5.7 (95% CI: 7.4 to 10.1)	7.6 ± 5.5 (95% CI: 6.3 to 8.9)	−1.2 ± 4.5 (95% CI: −2.3 to −0.1)	−0.4 ± 93.1 (95% CI: −22.8 to 21.9)	**0.018**
**Part III** (motor symptoms)	28.6 ± 13.9 (95% CI: 25.2 to 31.9)	25.2 ± 14.0 (95% CI: 21.9 to 28.6)	−3.3 ± 7.7 (95% CI: −5.2 to −1.5)	−8.3 ± 35.5 (95% CI: −16.8 to 0.2)	**<0.001**
**Part IV** ^a^ (motor complications)	4.4 ± 1.7 (95% CI: 3.9 to 4.8)	3.0 ± 2.0 (95% CI: 2.5 to 3.5)	−1.3 ± 1.7 (95% CI: −1.7 to −0.9)	−31.8 ± 44.1 (95% CI: −42.3 to −21.3)	**<0.001**
**Total** score	49.2 ± 20.2 (95% CI: 44.3 to 54.0)	43.5 ± 20.9 (95% CI: 38.5 to 48.5)	−5.7 ± 11.4 (95% CI: −8.4 to −2.9)	−11.0 ± 24.4 (95% CI: −16.8 to −5.1)	**<0.001**

Data are presented as mean ± SD and 95% confidence intervals. Negative values indicate a reduction, which equals improvement. Missing data (^a^ n = 1). CI, confidence interval; MDS-UPDRS, Movement Disorder Society-Unified Parkinson’s Disease Rating Scale; SD, standard deviation.

## Data Availability

The raw data supporting the conclusions of this article will be made available by the authors upon request due to proprietary information.

## Supplementary Materials

The following supporting information can be downloaded at https://www.mdpi.com/article/10.3390/brainsci15050532/s1. Table S1: Differences in frequency distributions of impact of fluctuations (classified according to MDS-UPDRS Part IV), between baseline and month 3; Table S2: Incidence of opicapone-related adverse events.

## Abbreviations

The following abbreviations are used in this manuscript:

AE	Adverse event
CGI-C	Clinician Global Impression of Change
CGI-S	Clinical Global Impression Severity
CI	Confidence Interval
COMT	Catechol O-methyltransferase
IQR	Interquartile range
LEDD	Levodopa equivalent daily dose
MAOI	Monoamine oxidase inhibitors
MDS-UPDRS	Movement Disorder Society-sponsored revision of the Unified Parkinson’s Disease Rating Scale
NMSS	Non-Motor Symptoms Scale
PGI-C	Patient Global Impression of Change
PD	Parkinson’s disease
PDQ-8	Parkinson’s Disease Questionnaire-8
QoL	Quality of life
SD	Standard deviation
WOQ-19	Wearing-off questionnaire
