# Correction: López-Manzanares et al. Real-World Use of COMT Inhibitors in the Management of Patients with Parkinson’s Disease in Spain Who Present Early Motor Fluctuations: Interim Results from the REONPARK Study. *Brain Sci.* 2025, *15*, 532

**DOI:** 10.3390/brainsci15111225

**Published:** 2025-11-14

**Authors:** Lydia López-Manzanares, Juan García Caldentey, Marina Mata Álvarez-Santullano, Dolores Vilas Rolán, Jaime Herreros-Rodríguez, Berta Solano Vila, María Cerdán Sánchez, Tania Delgado Ballestero, Rocío García-Ramos, Ana Rodríguez-Sanz, Jesús Olivares Romero, José Blanco Ameijeiras, Isabel Pijuan Jiménez, Iciar Tegel Ayuela

**Affiliations:** 1 Movement Disorders Unit, Department of Neurology, University Hospital La Princesa, 28006 Madrid, Spain; 2 Neurology Service, Hospital Quirónsalud Palmaplanas and Clínica Rotger, 07010 Palma de Mallorca, Spain; 3 Department of Neurology, University Hospital Infanta Sofia, 28702 Madrid, Spain; 4 Department of Neurology, University Hospital Germans Trias i Pujol, 08916 Barcelona, Spain; 5 Movement Disorders Unit, Department of Neurology, University Hospital Infanta Leonor, 28031 Madrid, Spain; hrinvest@hotmail.com; 6 Department of Neurology, Hospital Josep Trueta i del Santa Caterina de Salt, 17007 Girona, Spain; 7 Department of Neurology, University Hospital Santa Lucia, 30202 Cartagena, Spain; 8 Neurology Service, Hospital Parc Taulí, 08208 Barcelona, Spain; 9 Department of Neurology, Hospital Clínico San Carlos, 28040 Madrid, Spain; 10 Department of Neurology, University Hospital La Paz, 28046 Madrid, Spain; anarods@hotmail.es; 11 Neurology Service, University Hospital Torrecárdenas, 04009 Almería, Spain; 12 Medical Department, Laboratorios Bial, S.A., 28027 Madrid, Spain

## Text Correction

There was an error in the original publication [1]. In the Introduction Section, the following referenced sentence contains a misinterpretation: “Moreover, recent findings have indicated the benefit of adding opicapone at 50 mg to an additional 100 mg of levodopa in reducing OFF times in a cohort with early fluctuations [19]”. The sentence incorrectly suggests that the benefit arises from combining opicapone 50 mg with an additional 100 mg of levodopa, thus attributing to the referenced work a statement that differs from their original text. The correct interpretation is that opicapone 50 mg was more effective than an additional 100 mg of levodopa in reducing OFF time.

A correction has been made to the 5th paragraph of the Introduction Section. 

The revised paragraph reads as follows: “Moreover, recent findings have indicated that adding opicapone 50 mg is more beneficial than adding an additional 100 mg of levodopa in reducing OFF times in a cohort with early fluctuations [19].”

The authors state that the scientific conclusions are unaffected. This correction was approved by the Academic Editor. The original publication has also been updated.
