# Supplementary material for: Real-World Use of COMT Inhibitors in the Management of Patients with Parkinson’s Disease in Spain Who Present Early Motor Fluctuations: Interim Results from the REONPARK Study
Source: Brain Sci. 2025 May 21;15(5):532. doi: 10.3390/brainsci15050532 (PMC12110095; doi:10.3390/brainsci15050532)
Supplement: Supplementary file 1 [file brainsci-15-00532-s001.zip › brainsci-3598110-supplementary.pdf]

Supplementary tables

Supplementary Table S1. Differences in frequency distributions of impact of fluctuations (classified according to MDS-UPDRS Part IV), between baseline and month 3

	**Month 3***				
**Baseline**	No impact	Slight	Mild	Moderate	Total
No impact	6 (8.8%)	0	1 (1.5%)	0	7 (10.3%)
Slight	15 (22.1%)	8 (11.8%)	2 (2.9%)	2 (2.9%)	27 (39.7%)
Mild	8 (11.8%)	9 (13.2%)	6 (8.8%)	0	23 (33.8%)
Moderate	2 (2.9%)	3 (4.4%)	4 (5.9%)	2 (2.9%)	11 (16.2%)
Total	31 (45.6%)	20 (29.4%)	13 (19.1%)	4 (5,9%)	68 (100%)

*p< 0.001 vs baseline (McNemar test)
MDS-UPDRS, Movement Disorder Society-Unified Parkinson's Disease Rating

Supplementary Table S2. Incidence of opicapone-related adverse events

Event	Number of cases
Dyskinesia	4 (5.7)
Nervousness	2 (2.9)
Restlessness	2 (2.9)
Somnolence	2 (2.9)
Delusion	1 (1.4)
Abdominal pain	1 (1.4)
Insomnia	1 (1.4)
Confusional state	1 (1.4)
Paraesthesia	1 (1.4)
Asthenia	1 (1.4)
Balance disorder	1 (1.4)
Dizziness	1 (1.4)
Nausea	1 (1.4)
Pollakiuria	1 (1.4)
Feeling abnormal	1 (1.4)
Mental impairment	1 (1.4)

Data are presented as the number of cases divided by the total number of AEs recorded (69).
MedDRA Version 26.0 used
